# Virtual care models for cancer survivorship

**DOI:** 10.1038/s41746-020-00321-3

**Published:** 2020-09-02

**Authors:** Quynh Pham, Jason Hearn, Bruce Gao, Ian Brown, Robert J. Hamilton, Alejandro Berlin, Joseph A. Cafazzo, Andrew Feifer

**Affiliations:** 1 Centre for Global eHealth Innovation, Techna Institute, University Health Network, Toronto, ON Canada; 2 Institute of Health Policy, Management and Evaluation, Dalla Lana School of Public Health, University of Toronto, Toronto, ON Canada; 3 Faculty of Medicine, Memorial University of Newfoundland, St. John’s, NL Canada; 4 Division of Urology, Department of Surgery, University of Toronto, Toronto, ON Canada; 5 Division of Urology, Niagara Health, Niagara, ON Canada; 6 Faculty of Health Sciences, McMaster University, Hamilton, ON Canada; 7 Department of Surgical Oncology, Princess Margaret Cancer Center, University Health Network, Toronto, ON Canada; 8 Department of Radiation Oncology, University of Toronto, Toronto, ON Canada; 9 Radiation Medicine Program, Princess Margaret Cancer Centre, University Health Network, Toronto, ON Canada; 10 Institute for Better Health, Trillium Health Partners, Mississauga, ON Canada

**Keywords:** Translational research, Health services, Cancer models

## Abstract

Virtual care models for cancer survivorship are needed to support patients living with the chronic effects of cancer treatment, while increasing health system capacity. Characteristics that may be critical to their success have not been adequately studied. This scoping review summarizes previous efforts to virtualize survivorship care to inform future innovations in the field. Four databases were searched for articles published before January 2020, and 24 articles that met selection criteria were included in this analysis. Rationale for pursuing virtual models of care shared two common objectives: (1) the need for sustainable survivorship care, and (2) the opportunity to improve survivorship outcomes. Breast cancer (*N* = 10) and prostate cancer (*N* = 4) were the most targeted cancers for virtual survivorship care. The implemented technologies included web platforms (*N* = 15), telephone calls (*N* = 12), and smartphone or tablet applications (*N* = 5). A variety of healthcare professionals were effectively involved in the provision of virtual care. Future virtual care models may benefit from integrating with existing health systems and services, repurposing common technologies, involving allied health professionals, and engaging patients and caregivers from diverse communities in the design of virtual services.

## Introduction

Contemporary trends in cancer survivorship demand effective and sustainable models of post-treatment survivorship care^1^. The most acute of these trends is disease prevalence; 18 million North Americans live with cancer^2^, a number that is expected to increase dramatically over the next two decades^3^. Improvements in cancer screening and therapy have increased survival rates in Canada to 95% for prostate cancer, and 87% for breast cancer, with the number of surviving patients increasing correspondingly^4,5^. However, survivors are often left with myriad post-treatment functional impairments and psychosocial and mental health challenges that negatively impact their quality of life^6,7^.

Cancer survivors are operationally defined as “patients who have completed primary cancer treatment and have no evidence of disease”^8^. Conventional post-treatment cancer follow-up comprises in-person visits with specialists at pre-specified intervals^9^. These fixed protocols are not necessarily well-suited to address patient needs in a timely and accessible manner^10,11^. The scope of conventional specialist visits is largely focused on assessing the risk of cancer recurrence and the medical consequences of cancer and its treatment. Despite best intentions, the current model of care has limited capacity and time to provide comprehensive follow-up that meets endorsed survivorship practice guidelines^12–15^. These systemic issues suggest that current models of survivorship care were established in a different era of cancer survival and are no longer adequate to address the chronic and complex survivorship needs of modern patients. A redesign of existing post-treatment follow-up care pathways is needed to increase health system capacity and to better support patients living with the chronic effects of cancer and its treatment.

Recently, virtual care in cancer follow-up has emerged as a plausible means to deliver and receive care. Broadly defined as any remote interaction between patients and healthcare providers using technology to enhance the quality and effectiveness of care^16^, virtual cancer care models exploit technological innovation to deliver integrated, stratified, and tailored survivorship care to patients who are at low risk of recurrence^17^. Successes in virtualizing survivorship care have been realized through genitourinary telemedicine clinics, which demonstrated that the remote delivery of urologic care is safe, cost-effective, and yields high patient satisfaction^18^. A recent multi-center evaluation of a remote prostate cancer surveillance program implemented in the United Kingdom (*N* = 627) showed comparable clinical outcomes and lower costs when compared to traditional follow-up care, with patients favouring off-site and on-demand visits^19,20^. In Canada, virtual breast cancer specialist follow-up visits delivered through video and email have been explored and found to be acceptable for a subgroup of survivors with non-recurrence related needs^21^.

Although virtual services for cancer follow-up have garnered interest for their potential to alleviate pressure on healthcare services and better meet survivors’ long-term needs, the components that are critical to their success have not been systematically studied. Little knowledge exists regarding the optimal roles and responsibilities of providers and patients in these models, nor the range of services that can be safely delivered using technology. The breadth of virtual care models being deployed as part of survivorship research or standard of care has not been systematically cataloged. We propose that there is a benefit to understanding “what works for whom in which circumstances”^22^ to advance the effective application of these models across oncological contexts and improve them over time. Thus, the aim of this scoping review was to describe how virtual care models for cancer survivorship have been designed in the past, so as to surmise characteristics that may be critical to their success in the future.

## Results

### Screening

**Fig. 1:**
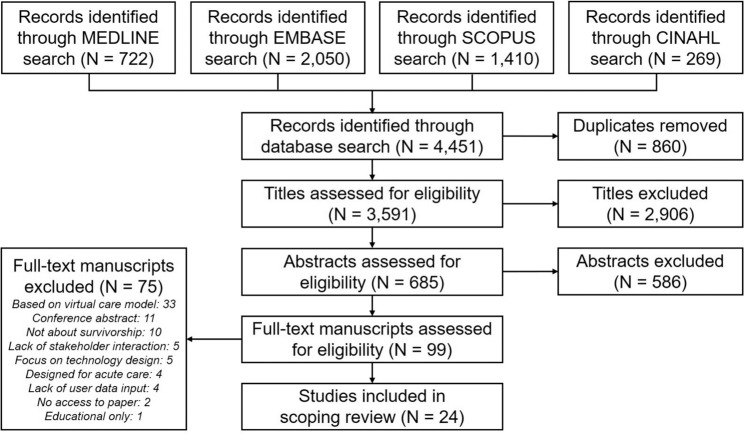
Flow diagram summarizing study selection.

A search of four databases returned a total of 4451 articles (722 MEDLINE, 2,050 EMBASE, 1,410 SCOPUS, 269 CINAHL). Deduplication reduced the number of unique articles to 3591. The title review identified 685 articles worthy of further consideration, of which 99 were found to meet the established criteria in the abstract review. Based on independent assessment of the full-text manuscripts by two members of the research team, 28 articles were selected for inclusion. Four more articles were excluded following discussions between the two reviewers, resulting in a final total of 24 articles selected for inclusion in the scoping review^20,23–45^. Full-text manuscripts were mostly excluded due to a lack of discussion on the provision of virtual care by provider to patient (*N* = 33), publication as a conference abstract (*N* = 11), or a lack of focus on survivorship (*N* = 10). The table summarizing the characteristics of the included studies can be found in Supplementary Data 1. The study selection process is summarized using a PRISMA-ScR flow diagram in Fig. 1.

### Study characteristics

A total of 10 articles described a study protocol and 14 articles presented results of a completed study. The average sample size of the selected studies was 307 participants. Study durations ranged from 4 weeks to 1 year. The most common primary outcomes related to intervention feasibility, fidelity, or adoption (*N* = 7); quality of life (*N* = 6); and physical fitness or weight loss (*N* = 4). Other targeted outcomes related to pain, anxiety, and self-efficacy. A single study focused on emergency department presentations. Nine studies reported ethnicity data of the study participants.

### Virtual care model characteristics

The emergent virtual care models targeted a variety of cancers including breast (*N* = 10); prostate (*N* = 4); pediatric (*N* = 3); and gynecologic cancer (*N* = 3). Seven of the interventions targeted three or more different adult cancer types. Examples of technology implemented in the selected studies included web platforms (*N* = 15), phone calls (*N* = 12), smartphone or tablet applications (*N* = 5), and fitness trackers or smart weight scales (*N* = 5). Most interventions were delivered entirely remotely (*N* = 18), while others involved remote care in addition to an in-person service (*N* = 6). In terms of healthcare providers, the virtual care models often involved a behavioral health provider (*N* = 11), a nurse (*N* = 9), a physician (*N* = 3), or a dietitian (*N* = 2). A total of 15 interventions included training for providers, while only four studies reported involvement of informal caregivers in intervention design or deployment.

### Breast cancer survivorship models

A total of ten studies included an intervention specifically targeted at breast cancer survivorship. Four of these studies focused on quality of life as the primary outcome. Galiano-Castillo et al. studied an 8-week internet-based tailored exercise program with the option for instant messaging and video calls with research staff. When compared to a control group, the telerehabilitation group saw significant improvements in global health status, physical and cognitive function, arm symptoms, pain severity, and pain interference^23^. Kimman et al. found that nurse-led telephone follow-up in the first year following breast cancer treatment yielded similar improvements to patient outcomes (i.e., quality of life, role functioning, emotional functioning, anxiety) when compared to traditional follow-up visits^24^. Krusche et al. are currently assessing the quality-of-life impacts of an intervention to support increased physical activity, regulation of difficult emotions, maintenance of a healthier diet, and weight management in breast, colorectal, and prostate cancer survivors^25^. Similarly, Anderson et al. are studying the impact of a program comprising an interactive journal, web interface, and virtual nurse-led health consultations on women recently treated for breast, blood, or gynecological cancer^26^.

Two protocols are currently targeting improvements in physical fitness and/or weight loss amongst breast cancer survivors. Ritvo et al. are assessing a multifaceted intervention comprising telephone-based coaching sessions, a wearable fitness tracker and a smartphone-based health tracking software^27^. Similarly, Reeves et al. are currently evaluating the efficacy of a telephone-delivered weight loss intervention focused on targeted dieting and physical activity^28^.

Four additional breast cancer interventions targeted alternative primary outcomes. Visser et al. tested a tablet application that enabled video-based group medical consultations and direct communication with a clinical nurse specialist. The intervention failed to improve the primary outcomes of distress and empowerment levels when compared to a control group, a result that the researchers attributed to already low levels at baseline^29^. Quintiliani et al. assessed the feasibility of an intervention comprising self-monitoring SMS messages, a fitness tracker, a smart weight scale, and phone sessions with a trained counselor. The pilot study reported high levels of engagement in all aspects of the intervention, as well as general improvements in weight, fruit and vegetable intake, and physical activity^30^. Two additional studies evaluated the effectiveness of technology-supported cognitive behavioral therapy in mitigating anxiety, depression^31^, and fatigue^32^ amongst breast cancer survivors. In the latter of the two studies, the intervention group reported improved fatigue scores, functional impairment, psychological distress, and quality of life when compared to a control group^32^.

### Prostate cancer survivorship models

In addition to the aforementioned protocol by Krusche et al.^25^, three additional articles have described virtual care models for prostate cancer survivors. Frankland et al. operationalized a program in which patient-reported outcome measures (PROMs) submitted via a web platform, as well as prostate-specific antigen (PSA) transferred directly from the lab, could be monitored remotely by support workers and uro-oncology clinical nurse specialists. In place of regular follow-up visits, the program enabled appointments to be booked on an as-needed basis by either the care team or the patient. With a reduction in per-participant costs when compared to the control group, the intervention group demonstrated improvements in unmet survivorship needs, activation of self-management, quality of life, psychological well-being and satisfaction with care^20^. Pham et al. are currently evaluating the adoption of a smartphone application in which patients can check their PSA, submit monthly PROMs relating to their prostate cancer-specific quality of life, and access a feed of educational content and survivorship-related social events. All patient-submitted data are accessible via a clinician dashboard by an assigned urologist, who is also alerted to worrisome data entries that may warrant intervention^33^. Song et al. are currently assessing the quality-of-life impacts of a web-based intervention for prostate cancer survivors and their partners, which offers educational modules, post-module assignments, a moderated support forum, meetings with a health educator, and online resources for symptom tracking^34^.

### Pediatric cancer survivorship models

Three identified articles presented a survivorship intervention for childhood or adolescent cancers. Jibb et al. developed a smartphone application for adolescents to characterize their cancer-related pain using a validated questionnaire and receive personalized self-management recommendations. In the event of severe pain, an email alert would also be sent to a registered nurse who could contact the patient. The intervention was well-accepted by participants, who demonstrated significant improvements in both pain intensity and quality of life in comparison to baseline. However, intervention fidelity was limited by technical challenges and delayed nurse contact in response to alerts^35^. Maurice-Stam et al. assessed the feasibility of an online cognitive behavioral therapy group intervention for adolescent cancer survivors. The developed website offered a secure chat room for weekly sessions with a pediatric psychologist, a list of homework, and educational resources for family members. The study reported low dropout rates and high levels of satisfaction amongst both patient and clinician participants^36^. Signorelli et al. are evaluating a nurse-led virtual care model for childhood cancer survivors consisting of an initial virtual consultation with a nurse, case review by a multidisciplinary team, a second virtual consultation with a nurse to discuss the results of the case review, and access to an electronic survivorship care plan^37^.

### Other cancer survivorship models

Virtual care interventions have also been described for colorectal and gynecologic cancers. Reese et al. evaluated the feasibility of a program to address the intimacy and sexual concerns of colorectal cancer patients and their partners. The intervention, which comprised 50-min telephone-based sessions incorporating techniques from sex and couple therapy, was found to be helpful by most participants^38^. Haggerty et al. assessed the efficacy of two separate virtual care models for endometrial cancer survivors with obesity. The first intervention involved the combination of a smart weight scale and telephone-based weight-loss counseling. The second group received a conventional scale and 3–5 supportive SMS messages daily. Neither intervention led to increased weight loss when compared to the control group. The telephone-based group reported increased physical activity and improved cancer-related body image compared to the SMS-based group, as well as improved sexual functioning compared to the control group^39^. Wenzel et al. assessed the effect of a psychosocial telephone-based counseling intervention, comprising five weekly sessions and a 1-month booster, on quality of life in cervical cancer survivors. The intervention group reported improvements in depression, gynecologic concerns, and cancer-specific concerns at 4 months, with the latter two findings being maintained at 9 months^40^.

Five interventions have been described for all survivors regardless of their cancer type. Braun et al. evaluated feasibility and acceptability of technology-based motivational interviews via email, telephone, SMS messages, or Skype with registered dietitian nutritionists. Users of the intervention set more goals, lost more weight, and reported improved quality of life when compared to a control group^41^. Zernicke et al. provided an online synchronous mindfulness-based cancer recovery program for underserved cancer survivors. The program was feasible in terms of both recruitment and retention. The intervention group reported improvements in mood disturbance, stress, spirituality, and mindfulness when compared to a group randomized to “wait for the next available program”^42^. Gell et al. evaluated a 4-week intervention for cancer survivors comprising an activity tracker, tailored SMS messages, and brief health coaching session. Mean daily step counts and weekly minutes of activity were maintained post-intervention when compared to baseline levels. Self-regulation and fatigue levels were also improved when compared to baseline^43^. Tamminga et al. are currently evaluating a web-based stepped-care intervention for cancer survivors returning to work. The program involves two steps: (1) provision of tailored information on cancer and work, and (2) self-management of problems inhibiting successful return to work^44^. Last, Girgis et al. are assessing a web-based platform that allows cancer survivors to submit PROMs, which are directly transmitted to their provider. Patient-reported data are used to alert clinicians of patients with unresolved issues, as well as to provide the patient with tiered self-management resources. The ongoing study will analyze the effect of virtual care on emergency department visits, chemotherapy adherence, and health service referrals^45^.

## Discussion

This review reveals important information regarding the characteristics of virtual care models for cancer survivorship. Virtual cancer survivorship care is an emerging area of research, with nearly half of the selected studies describing a protocol of an ongoing intervention. The high prevalence of novel interventions highlights the nascency of virtual cancer survivorship as a field of study and practice. Rationale for pursuing virtual models of care varied across clinical and research contexts but shared two common objectives: (1) the need for sustainable survivorship care, and (2) the opportunity to improve survivorship outcomes. Many studies cited new policies and practice guidelines that advocated for replacing the traditional ‘one size fits all’ model of scheduled follow-up care with innovative programs tailored to cancer patients at low risk of recurrence^46,47^. While not explicit in their endorsement of any one care modality, these top-down mandates to maximize both healthcare investments and patient experiences were interpreted by researchers to be aligned with virtual care. We noted that researchers never led with a “digital-first” argument for virtualizing services, despite showing an interest in technological innovation and highlighting it as a strength of their approach^48^. Instead, they positioned technology as a practical means to increase provider capacity and improve access to care. Our findings highlighted that virtual care models are being developed for survivors of various cancers, with breast and prostate cancer being the pathologies of greatest apparent interest. Targeting the two most common cancers in North American men and women makes practical sense given the intent for virtual care to significantly reduce pressure on healthcare services.

A wide variety of healthcare providers were involved in the delivery of virtual services. Nurses, therapists, counselors, and dietitians all provided care within these virtual models, some under the supervision of more specialized clinicians. This finding supports mounting evidence of the viability and effectiveness of involving allied health professionals in cancer survivorship care^49,50^. Nurse-led survivorship models in the United States and United Kingdom have been proven to be clinically beneficial and cost-effective, and have yielded high satisfaction among patients and supporting staff. Systematic reviews and practice guidelines recommend that better integration of nursing roles in survivorship services will improve quality of care, patient experiences, and health outcomes, all while maintaining or reducing the use of other healthcare services to promote systems-wide cost savings^51–54^.

Numerous studies failed to mention provider training prior to deployment of their respective virtual care models. This lack of training may be problematic, as many interventions were largely dependent on the provider’s ability to interact with the deployed technology. Moreover, few studies mentioned the number of providers that were tasked with provision of each virtual care model, which in turn prohibited calculation of the patient-to-provider ratio. Such information is critical when considering the potential scalability and sustainability of a digital health intervention^55^.

Several studies failed to report with granularity the social determinants of health that impacted their studied patient populations. For example, the majority of studies evaluated a virtual care model amongst a patient population composed primarily of Caucasian participants. As survivors from minority communities are more likely to face complications following cancer treatment^56^, it is imperative that all survivorship interventions be designed in a culturally compassionate manner. Accordingly, cultural diversity should be pursued and reported in the validation of virtual care models. Only four interventions mentioned incorporation of informal caregivers (e.g., partners, parents, children) within the virtual circle of care. Given the accumulation of evidence that “cancer is a family affair”^57^, survivorship researchers should strongly consider involving informal caregivers in the future design, development and deployment of such programs.

The majority of the described interventions involved solely remote interactions with patients, while a minority used a blended model encompassing both remote and in-person components. Most remote care models focused on improving patient self-management or completing technology-based counseling sessions, both of which were shown to be effective in improving patient outcomes. A variety of blended models were evaluated, such as initial in-person visits followed by remote management, remote management with in-person visits as needed, and remote management in conjunction with regular in-person visits. The appropriate mode of delivery for a given virtual care model appeared to be largely situation-dependent. For example, fully remote programs were appropriate for the delivery of counseling or educational content, whereas blended models were better-suited for interventions detecting potential complications that may require clinical intervention.

The majority of technologies used to power the virtual models of survivorship care were not technologically advanced. Many interventions used simple methods of communication such as websites, telephone calls, and SMS messaging. Interventions were often delivered using multiple consumer technologies that were combined with custom content to form a fit-for-purpose solution. Studies appeared to prioritize the clinical aspect of the described intervention rather than the supporting technology. Very few studies reported integration of the developed intervention with the health system. This lack of integration generally allows for faster scaling of the intervention, but ultimately results in a ceiling effect in terms of its clinical applicability, sustainability, and effect on patient–clinician relationships^55,58^. The benefits of health system integration were demonstrated by Frankland et al., whose nurse-led virtual prostate cancer survivorship clinic was found to decrease per-patient cost, while also improving survivorship needs. This virtual survivorship model has since been adopted as part of routine cancer care in four hospital trusts in the United Kingdom^59^.

The length of the research phase in the identified studies varied widely from 4 weeks to 1 year. This wide range fails to elucidate the appropriate amount of time for patients to be enrolled in virtual care models. For studies of short duration, it is also difficult to ascertain whether patients and providers would engage with these virtual models long enough to form strong patient–clinician relationships and to derive sustained outcomes. Furthermore, very few studies reported a plan for transition out of the research phase into clinical deployment. Implementation research is needed to identify how patient and provider interest in these virtual care models can be sustained beyond a short trial period to provide enduring value^60^. Along with the healthcare integration discussed previously, effective transition of virtual care models into clinical practice requires careful consideration of payment models, process design, and technical support^58^. Though many payment models currently fail to adequately incentivize the provision of virtual care^61^, fee codes directly related to such services have launched specifically as a result of the COVID-19 pandemic^62,63^. While it remains to be seen whether these services will be maintained and remunerated beyond the current crisis, their rapid deployment suggests that health systems can bear the cost of innovating cancer care when motivated.

The selected studies generally targeted *soft* outcomes such as feasibility and quality of life^64^. The only study to target a *hard* primary endpoint was the protocol by Girgis et al, which is currently evaluating the effect of a virtual survivorship service on emergency department presentations. The focus on soft outcomes correlates with previous reviews of the digital health literature^65^, and is likely attributable to the lack of clinical deployment and general immaturity of the digital health field. As a direct result of low digital health adoption rates and limited integration in clinic workflows, it is currently difficult (though not impossible) for digital health solutions to effect change in hard outcomes such as hospitalizations, morbidity, and mortality^66^. Girgis et al. may have been motivated to target hard outcomes with their virtual care model because (1) they had already demonstrated the feasibility and acceptability of their model in their target population^67^, and (2) components of their model had previously been integrated into relevant hospital point-of-care systems^45^. With continued development and validation of virtual care models that effectively integrate into the lives of both patients and clinicians, improvements in these hard outcomes are likely to follow^68,69^. However, expectations of their effect size should be measured as the survivorship delta is small and will require large studies to demonstrate effectiveness.

While virtual care models hold the promise of improved medical and psychosocial outcomes, technological innovations are developed at a rate that often exceeds information privacy, security, and safety regulations^70^. Almost all studies identified a consent process during recruitment of patients. As part of the consent process, physicians and patients involved in virtual care should understand the limitations and risks associated with the intervention and agree to be accountable for protecting personal health information and safety. Privacy breaches and preventable adverse medical outcomes can result in significant medical–legal difficulties and compromise patient–physician relationships. Care providers should ensure that security safeguards are in place, such as locking devices when not in use and employing privacy settings appropriate to the local regulatory body (e.g., password protection, encryption)^71^. Furthermore, although virtual care models may triage safety concerns in PROMs to notify care providers^35^, a back-up plan should be available if the device, technology or model were to fail. Rapid re-entry pathways to on-site follow-up clinics or the emergency department should be made available to patients as needed.

From a safety and medical–legal perspective, clear medical documentation is crucial to protect patients and providers. Linking a virtual care model directly to an electronic medical record or government data repository may assist in continuity of care as the patient transitions between outpatient and emergency/inpatient medical settings^45^, while also maintaining adequate health record documentation. Ultimately, clearly defining where legal responsibility lies, setting expectations, and creating clear medical documentation may assist patients and physicians in making informed decisions about adopting a virtual care model.

This study is not without limitations. As a result of the implemented scoping review methodology that aims to map the field of study, the researchers did not evaluate the underlying quality of each individual study. Similarly, the study did not assess which of the individual virtual care models is most likely to improve patient outcomes. The literature search was also limited to articles published in English, as well as those indexed in the four selected databases (i.e., MEDLINE, EMBASE, SCOPUS, CINAHL). Due to the resource limitations, only one reviewer conducted the search and screened titles against eligibility criteria, thereby potentially introducing bias prior to the abstract and full manuscript review by two reviewers. A further limitation was that many of the included manuscripts described study protocols. The lack of implementation outcomes available for analysis prevented a definitive identification of characteristics critical to the success of operationalizing virtual models for cancer survivorship in clinical practice. Finally, the decision to not exclude studies based on clinical pathologies or patient populations meant that selected studies were heterogeneous in scope, thereby limiting the generalizability of insights on what will work or not work in a virtual cancer survivorship model.

Virtual care models are actively being researched as a means of improving cancer survivorship care and increasing health system capacity. This scoping review abstracts the characteristics of virtual care models for cancer survivorship that have been designed to date, so as to better understand factors that may contribute to the success of future interventions. Past virtual survivorship models have generally repurposed common technologies (e.g., telephones, video conferencing software) rather than building new solutions; such simple and accessible tools may enable more equitable virtual care for cancer survivors. Previous interventions have also involved a variety of allied health professionals, allowing patients to be paired with the appropriate provider while optimizing resource allocation. We noted that the selected articles rarely discussed the inclusion of patients, providers, and caregivers in virtual care model design and development. Moreover, few articles described the socioeconomic status and ethnoracial considerations of the studied patient populations. Future interventions may benefit from incorporating the needs and lived experiences of all members in the circle of care, especially those from minority communities. Last, most virtual care models were not integrated with existing health systems and services. Improved integration may enhance the clinical effectiveness and sustainability of virtual care models for cancer survivorship.

## Methods

### Study design

A scoping review was conducted to address the following research question: *What are the characteristics of virtual care models for cancer survivorship?* The review was guided by scoping review methodology proposed by Arksey & O’Malley^72^, as well as the Preferred Reporting Items for Systematic reviews and Meta-Analyses extension for Scoping Reviews (PRISMA-ScR)^73^. The completed PRISMA-ScR checklist used in this analysis is included in Supplementary Table 1.

### Study strategy

A search strategy was devised to capture protocols or full evaluations of virtual care models for cancer survivorship. As suggested by a librarian based on the focus of the review, the investigators searched MEDLINE, EMBASE, SCOPUS, and CINAHL for primary articles published before January 2020. The scope of the search was not restricted to a particular chronic cancer or virtual care technology. A test search was conducted on MEDLINE to make sure that the strategy returned a series of pre-identified articles that were deemed appropriate. The developed search strategy is included in Supplementary Reference 1.

### Inclusion and exclusion criteria

The inclusion criteria informing the selection of relevant articles were as follows:

- The article described an evaluation or a protocol for an evaluation of a virtual care model for managing cancer survivorship;
- The article was published and accessible in English;
- The intervention enabled the provision of technology-mediated care from a provider to a patient;
- The intervention enabled either synchronous or asynchronous communication between patients and providers;
- The intervention required interaction from both patients and providers;

Similarly, the exclusion criteria used in study selection were as follows:

- The article primarily described intervention technology design, development, or usability testing;
- The intervention was solely an appointment reminder service;
- The intervention involved only automated short message service (SMS) texts or an interactive voice response system;
- The intervention was designed for an acute context (e.g., post-operative care);
- The intervention was solely a support tool for a patient’s circle of care;
- The intervention did not require active or passive (e.g., sensor) data entry by the patient;
- The intervention only delivered educational information;

### Study selection

Article references emerging from the initial search were imported into the reference manager Mendeley v1.19.5 (Elsevier, Amsterdam). Deduplication was conducted to remove multiple entries of the same research article. A multi-step review process was undertaken to screen eligible articles. First, a research analyst independently reviewed each of the article titles and removed those that failed to meet the inclusion and exclusion criteria. Next, two reviewers (research analyst and lead author) independently assessed abstracts to further assess the relevance of the remaining articles to the research study. They then assessed full-text manuscripts for those articles meeting the established criteria in the abstract review. Any uncertainty regarding the relevance of certain articles was discussed amongst the two researchers until a consensus was reached.

### Data extraction

A standardized dataset was constructed to summarize information extracted from the identified articles. The following fields were extracted from the included articles; title, year, lead author, study phase, DOI, cancer type, primary provider, technology implemented, sample size, study duration, primary outcomes, age (mean and range), gender, ethnicity, caregiver involvement, provider training, and modality of virtual care (e.g., remote, remote with regular on-site visits). Data supporting the findings of this scoping review are available in this dataset and the included articles.

## Supplementary information

Supplementary Data 1

Supplementary Information

## Data Availability

All data generated or analyzed during this study are included in this article and its supplementary information files.
